# Supplementary material for: Causal Relationship between Aging and Anorexia Nervosa: A White-Matter-Microstructure-Mediated Mendelian Randomization Analysis
Source: Biomedicines. 2024 Aug 16;12(8):1874. doi: 10.3390/biomedicines12081874 (PMC11351342; doi:10.3390/biomedicines12081874)
Supplement: Supplementary file 1 [file biomedicines-12-01874-s001.zip › Supplementary Figure/Figure S8/Leaveoneout plot of MR analysis for TL to ubm-b-1608.pdf]

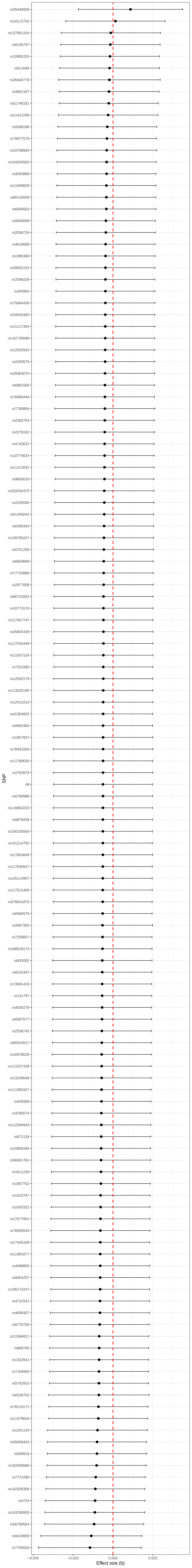

rs35446936
rs10112752
rs137901416
rs8105767
rs10905255
rs611646
rs35640778
rs3891167
rs61748181
rs11412296
rs9398196
rs79977579
rs10768683
rs144204502
rs3093888
rs11699829
rs80116508
rs6669563
rs9940099
rs2056726
rs4616688
rs1985369
rs28502153
rs7099229
rs450962
rs75664430
rs34550383
rs11117354
rs142730696
rs12925933
rs2293579
rs28363070
rs6881568
rs76666449
rs7790856
rs2282764
rs2276182
rs4743037
rs10774624
rs11212631
rs9600019
rs202034370
rs2230590
rs61405042
rs6590343
rs139795227
rs6751209
rs6659669
rs77732866
rs2977608
rs66731853
rs10773176
rs117407747
rs45604339
rs117034449
rs11557154
rs7221585
rs12932179
rs113525195
rs12412214
rs41304832
rs9955360
rs1957937
rs78491606
rs11769630
rs2763979
All
rs6790988
rs116863223
rs9878436
rs150150565
rs141214782
rs17803849
rs117630647
rs145114957
rs117512405
rs376641875
rs6584579
rs2967355
rs7209057
rs188918174
rs932002
rs8102497
rs73581419
rs131797
rs4530278
rs6587577
rs2538745
rs80324517
rs16978028
rs111527438
rs13230646
rs111950327
rs429358
rs3785074
rs112394943
rs871134
rs10805346
rs56061761
rs1611236
rs1907702
rs1023767
rs1003322
rs17677991
rs76065543
rs17445108
rs11991877
rs4498805
rs6054257
rs185174247
rs4731541
rs4695407
rs6776756
rs11584821
rs869785
rs1332941
rs7164950
rs5742915
rs6536702
rs76219171
rs11579626
rs1291143
rs59409453
rs939916
rs182059586
rs7772289
rs142426306
rs4724
rs143190905
rs56799554
rs9419958
rs7705526
SNP
−0.050
−0.025
0.000
0.025
Effect size (b)
